# Development of 3D-Printed Hydrogel Disks as Standardized Platform for Evaluating Excipient Impact on Metronidazole’s Antimicrobial Activity

**DOI:** 10.3390/pharmaceutics17060749

**Published:** 2025-06-06

**Authors:** Tomasz Gnatowski, Joanna Kwiecińska-Piróg, Tomasz Bogiel

**Affiliations:** 1 Department of Pharmaceutical Technology, Faculty of Pharmacy, Collegium Medicum in Bydgoszcz, Nicolaus Copernicus University in Toruń, 2 Jurasza Str., 85-089 Bydgoszcz, Poland; tomasz.gnatowski@cm.umk.pl; 2 Department of Microbiology, Faculty of Pharmacy, Collegium Medicum in Bydgoszcz, Nicolaus Copernicus University in Toruń, 9 Maria Curie-Skłodowska Str., 85-094 Bydgoszcz, Poland; j.kwiecinska@cm.umk.pl; 3 Department of Propaedeutics of Medicine and Infection Prevention, Faculty of Pharmacy, Collegium Medicum in Bydgoszcz, Nicolaus Copernicus University in Toruń, 9 Maria Curie-Skłodowska Str., 85-094 Bydgoszcz, Poland

**Keywords:** 3D printing (extrusion-based), antibacterial wound dressings, *Bacteroides fragilis*, drug delivery, hydrogels, metronidazole, personalized therapy, plasticizers, polyvinyl alcohol

## Abstract

**Background/Objectives**: Effective drug delivery systems require precise formulation and understanding of excipient impact on active pharmaceutical ingredient (API) stability and efficacy, as uncontrolled interactions can compromise outcomes. This study developed and validated a semi-solid extrusion (SSE) 3D printing method for polyvinyl alcohol (PVA)-based hydrogel disks with metronidazole (MET). These disks served as a standardized platform to assess excipient influence on MET’s antimicrobial activity, focusing on plasticizers (polyethylene glycol 400, glycerol, propylene glycol, and diethylene glycol monoethyl ether)—excipients that modify hydrogel properties for their application in printing dressing matrices—with the platform’s capabilities demonstrated using in vitro antimicrobial susceptibility testing against *Bacteroides fragilis*. **Methods**: Hydrogel inks based on PVA with added plasticizers and MET were prepared. These inks were used to 3D-print standardized disks. The MET content in the disks was precisely determined. The antimicrobial activity of all formulation variants was evaluated using the disk diffusion method against *B. fragilis*. **Results**: The incorporated plasticizers did not negatively affect the antimicrobial efficacy of MET against *B. fragilis*. All printed hydrogel matrices exhibited clear antimicrobial activity. The 3D-printed disks showed high repeatability and precision regarding MET content. **Conclusions**: SSE 3D printing is viable for manufacturing precise, reproducible MET-loaded PVA hydrogel disks. It provides a standardized platform to evaluate diverse excipient impacts, like plasticizers, on API antimicrobial performance. The tested plasticizers were compatible with MET. This platform aids rational formulation design and screening for optimal excipients in designed formulations and for various pharmaceutical applications.

## 1. Introduction

The development of effective and safe drug delivery systems is a cornerstone of modern pharmaceutical sciences, demanding a systematic approach to formulation design and a thorough understanding of the product and manufacturing process. As outlined in the ICH Q8 (R2) guideline on pharmaceutical development, quality should be built into the product by design (Quality by Design, QbD), which necessitates a comprehensive understanding of how active pharmaceutical ingredient (API) characteristics, excipient properties, and process parameters interact to define the final product’s quality and performance [1]. This is particularly critical for topical or locally acting drug products, where precise control over drug release and local concentration is paramount for therapeutic efficacy and minimizing potential adverse effects or the development of microbial resistance.

A significant clinical challenge where advanced drug delivery systems are urgently needed is the management of chronic wounds. These are a serious and growing global clinical problem. Their treatment is usually long-term, which significantly burdens healthcare systems and reduces the quality of life of patients [2]. A key factor complicating wound healing is bacterial infections, including those caused by anaerobic bacteria. These include *Bacteroides fragilis*—a Gram-negative, strictly anaerobic rod, often isolated from chronic wounds (including diabetic foot ulcers, pressure sores, venous leg ulcers), which is also responsible for their unpleasant odor [3]. Infections caused by *B. fragilis* and other anaerobes pose a serious therapeutic challenge due to increasing resistance to antibiotics and difficulties in diagnosis, they and also constitute a significant psychological and physical burden for the patient when the infection is accompanied by foul odor and pain [3,4].

Metronidazole (MET) is a chemotherapeutic drug from the nitroimidazole group, widely used in the treatment of infections caused by anaerobic bacteria and protozoa [5,6]. Its mode of action is based on the reduction of the nitro group in anaerobic conditions, which leads to the formation of cytotoxic metabolites that damage the DNA of microorganisms [6]. The choice of MET as a model substance in this study was dictated by the fact that resistance to this drug remains relatively low compared to other antibacterial substances used in the treatment of anaerobic infections, such as clindamycin or moxifloxacin [7], confirmed efficacy in wound treatment (including malodorous wounds) [4,8,9,10,11,12,13], good clinical tolerance, and the potential stability of this antimicrobial in the process of manufacturing hydrogel dressings [14]. Despite its established position in therapy of infections caused by anaerobic bacteria, the efficacy of MET, especially in the local treatment of chronic wound infections, may be limited by difficulties in achieving and maintaining an appropriate therapeutic concentration of a drug at the target site [15], underscoring the need for improved delivery strategies.

In the search for new therapeutic strategies, there is growing interest in modern systems, such as hydrogel dressings manufactured using the semi-solid extrusion (SSE) 3D printing technique, also known as hydrogel-forming extrusion (HFE) [16,17]. This is an additive manufacturing process consisting in precise, layered deposition of a semi-solid hydrogel “ink” in accordance with a digital model [16]. The key to the success of this method is the optimization of the rheological properties of the ink (viscosity, yield stress, thixotropy) and the printing process parameters (e.g., pressure, feed rate, nozzle geometry), which affects extrudability, printing accuracy, and shape fidelity [16,17]. Printed structures often require an additional stabilization step, e.g., by cross-linking (thermal, chemical, ionic, solvent evaporation), to obtain the desired structural and mechanical integrity. SSE/HFE technology offers significant advantages such as the possibility of working at room temperature, personalization of geometry for a specific patient, use of a wide range of materials (including biocompatible polymers), and precise control of the porous structure, which allows for the design of systems with controlled release of active substances. Thanks to this, SSE becomes a promising tool for creating advanced, personalized dressings and therapeutic systems [16,17]. Among the polymers used to create hydrogels, polyvinyl alcohol (PVA) is of particular interest in pharmaceutical and medical applications [18,19,20,21,22]. PVA is non-toxic, biocompatible, easy to process, and its properties can be easily modified [23,24,25]. It is used, among others, as a component of hydrophilic matrices of oral drug forms, transdermal systems, and hydrogel dressings [18,26,27,28,29,30,31,32].

The comprehensive development of such advanced, 3D-printed hydrogel dressings, aligned with QbD principles [1], is a multi-stage process. It encompasses not only the selection of the API and polymer but also the optimization of the formulation for suitable rheological properties for printing, desired final mechanical characteristics of the dressing, drug release profile, stability studies, sterilization methods, and the design of personalized geometries. The research presented in this manuscript forms part of a broader investigation into the development of 3D-printed metronidazole dressings. This particular study focuses on a foundational aspect: establishing and validating a platform to evaluate the impact of key excipients—specifically plasticizers—on critical quality attributes such as dose uniformity and antimicrobial activity of the API within precisely manufactured hydrogel matrices. A significant challenge in this field is the lack of standardized, reproducible methods for assessing how individual formulation components influence the critical quality attributes of 3D-printed drug products. Addressing this gap, this work hypothesized that SSE 3D printing can produce highly precise and reproducible MET-loaded hydrogel disks suitable as a standardized tool (platform) for such evaluations. It was further hypothesized that the addition of selected plasticizers, chosen for their potential beneficial effects on hydrogel properties, would not negatively affect MET’s antimicrobial activity against *Bacteroides fragilis* and might potentially modulate this activity, for instance, by influencing drug release. The development of such an evaluative platform, enabling rational formulation design, could contribute to therapies with improved efficacy, potentially aiding in mitigating the development of microbial resistance associated with suboptimal dosing.

Hydrogel formulations, in addition to the carrier polymer and the active substance (active pharmaceutical ingredient, API), often contain auxiliary substances, among which plasticizers play an important role [33]. Their addition is aimed at modifying the rheological properties [34], mechanical properties (e.g., improving elasticity) [35,36,37], and water absorption capacity. In addition, plasticizers can improve the processability of hydrogels, which is important, for example, in SSE printing processes [16,38]. Typical plasticizers include polyethylene glycols (PEGs) [39,40], glycerol (GL) [37,41,42,43], and propylene glycol (PG) [36,41]. This study also included diethylene glycol monoethyl ether (DEGEE), which, apart from its potential impact on the mechanical and rheological properties of the hydrogel, is used in topical preparations as a solvent, strong solubilizer, and penetration enhancer through the skin for active substances [44]. Its addition was considered because of previous reports of possible difficulties in reaching the site of action of locally administered MET [15].

Therefore, the primary aim of this study was to develop and validate a semi-solid extrusion 3D printing methodology for producing standardized PVA-based hydrogel disks containing MET, intended as a platform for formulation assessment in line with pharmaceutical development principles [1]. A further objective was to utilize this platform to systematically investigate the influence of the aforementioned plasticizers on the content uniformity and in vitro antimicrobial efficacy of MET within these 3D-printed matrices. This approach sought to establish a robust method for excipient screening and the optimization of hydrogel formulations for potential therapeutic applications.

## 2. Materials and Methods

### 2.1. Materials

Methanol (LiChrosolv, gradient grade) was purchased from Millipore (Burlington, MA, USA). Polyvinyl alcohol (PVA) (Mw 31,000–50,000, 98–99% hydrolyzed) and metronidazole (MET) were obtained from Sigma-Aldrich (St. Louis, MO, USA). Sodium acetate trihydrate (Ph. Eur.) was purchased from Riedel-de Haën (Seelze, Germany). Sodium hydroxide (analytical grade) and propylene glycol (PG) (analytical grade) were obtained from POCH (Gliwice, Poland). Brucella agar plates with 5% sheep blood, vitamin K, and hemin were supplied by GRASO (Starogard Gdański, Poland). Anaerobic atmosphere generators (GENBag) were purchased from bioMérieux (Marcy-l’Étoile, France). The bacterial strains used in this study included *B. fragilis* ATCC^®^ 25285™ and *Finegoldia magna* ATCC^®^ 29328™, both obtained from the American Type Culture Collection (ATCC, Manassas, VA, USA). Oxoid™ metronidazole antimicrobial disks (50 µg) were purchased from Thermo Fisher Scientific Inc. (Waltham, MA, USA). Syringe filters (nylon, 0.22 µm, Ø 25 mm) were supplied by Alfatec Technology (Łódź, Poland). Macrogol 400 (polyethylene glycol 400 (PEG 400), Ph. Eur. 6.0) was purchased from Caelo (Hilden, Germany). Diethylene glycol monoethyl ether (Transcutol P, DEGEE) was obtained from Gattefossé (Saint-Priest, France). Anhydrous glycerol (GL) (analytical grade) was obtained from POCH (Gliwice, Poland). Ultrapure water used in the preparation of solutions was obtained from a Synergy UV water purification system (Millipore, Burlington, MA, USA).

### 2.2. Study Design

In order to evaluate the developed MET hydrogels, a two-stage study was planned, including the analysis of MET content and antimicrobial activity. The main assumptions of the project are presented below:

Stage I—Optimization of MET content in disks for the assessment of its antimicrobial activity

1. A series of hydrogel inks differing in MET concentration were prepared.
2. From the obtained hydrogel inks, hydrogel matrices in the shape of microbiological disks were printed. MET-containing disks, which were assessed with the disk diffusion method, were obtained for content analysis and to determine at what content of the active substance the greatest changes in the growth inhibition zones of *B. fragilis* ATCC^®^ 25285™ appear.
3. On this basis, the optimal MET concentration was selected in the hydrogel ink and printed disk, guaranteeing the highest sensitivity of the method (large differences in the inhibition zones with small changes in MET concentration).

Stage II—Assessment of the impact of plasticizers on MET activity

1. Based on the results of Stage I, a series of hydrogel inks with a fixed, constant MET content was prepared, but differing in the type and/or the amount of plasticizers (polyethylene glycol 400 (PEG 400), propylene glycol (PG), diethylene glycol monoethyl ether (DEGEE), anhydrous glycerol (GL)).
2. Hydrogel matrices in the shape of microbiological disks were printed from the obtained hydrogel inks.
3. The final antimicrobial activity of MET-contained disks was assessed using the disk diffusion method (measuring the bacterial growth inhibition zones) and MET content analysis using the HPLC method. In this way, it was verified whether the presence of individual plasticizers affects the availability and activity of MET.

### 2.3. Preparation of Hydrogel Inks

Hydrogel inks were prepared according to the following procedure: in a 100 mL screw-capped bottle, approximately 25.0 g of water was weighed, and a magnetic stir bar was placed inside. The bottle was then sealed, placed in a water bath on a C-MAG HS 7 control magnetic stirrer (IKA, Staufen, Germany), and heated to 90 °C with continuous stirring. An accurately weighed amount of MET was added to the hot water, and once it had dissolved, polyvinyl alcohol (PVA) was introduced into the bottle under constant stirring. The contents were mixed for 2 h to ensure complete dissolution of the polymer. Next, the selected plasticizer (PEG 400, PG, GL, or DEGEE) was added to the PVA–MET solution and mixed for an additional 30 min. The resulting solution was then transferred to a 50 mL volumetric flask; any remaining solution in the bottle was rinsed out with hot water, and the rinses were also added to the flask. After cooling, the flask was filled up to 50 mL with water and thoroughly mixed on a shaker. Finally, the obtained hydrogel was transferred into 50 mL Falcon-type tubes (Sarstedt, Nümbrecht, Germany) and centrifuged at 4000× *g* for 15 min in an MPW–350R centrifuge (MPW MED. INSTRUMENTS, Warsaw, Poland) to remove any air bubbles.

Hydrogel inks varied in composition according to the objectives of each experimental stage. In Stage I, aiming to determine the optimal MET content in the printed disks, a series of formulations were prepared using the compositions listed in Table 1. In Stage II, designed to assess the impact of various plasticizers on MET activity, the hydrogel inks all contained the same MET concentration but differed in the type and ratio of the plasticizer, as indicated in Table 2. Following these steps, the hydrogel inks were ready for 3D printing, HPLC quantification, and antimicrobial evaluation as described in the subsequent sections.

### 2.4. Printing of Hydrogel Disks

#### 2.4.1. Design of Microbiological Disks

**Figure 1:**
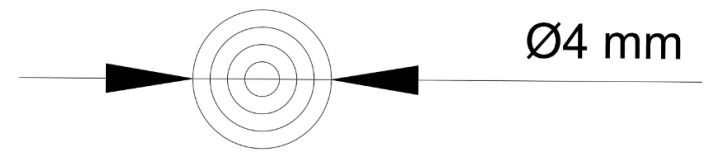
Microbiological disks design.

**Figure 2:**
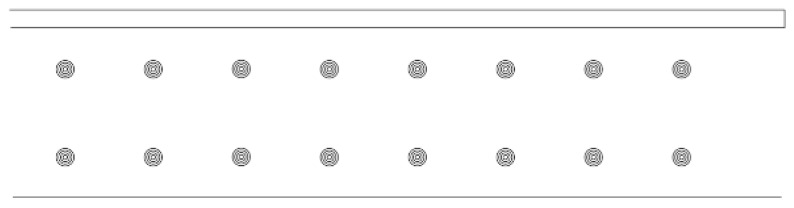
Arrangement of the designed disks on the printer table.

In microbiological method-based studies, 6 mm cellulose disks are traditionally used. To closely reproduce these dimensions via 3D printing, models were developed using AutoCAD 2020 (Autodesk, San Francisco, CA, USA). Owing to the tendency of hydrogel to spread upon extrusion, the designed disk diameter was reduced to 4 mm—defined by four concentric circles spaced 0.5 mm apart (Figure 1)—thereby yielding a final printed disk of 6 mm in diameter. Each batch of disks was printed in sets of 16, and their arrangement on the printer table, as well as their final appearance, are shown in Figure 2.

#### 2.4.2. Printing Parameters and Procedure

Models created in AutoCAD 2020 were saved in .DXF format and subsequently imported into Voxelizer 2.0 software, where they were converted into GCODE files according to the parameters specified in Table 3.

The printing parameters listed in Table 3 were largely established based on preliminary optimization studies aimed at ensuring consistent extrusion and print quality. Key fixed parameters were chosen as follows: a single layer (layer count: 1) was applied with a layer height of 0.1 mm, which was found to be optimal for free hydrogel flow and adhesion to the build plate. A constant print speed of 10 mm/s was used to maintain relatively low backpressure with an acceptable printing time, while a travel speed of 120 mm/s and a retraction height of 2 mm ensured rapid and clean movements between prints without dislodging the printed structures. The path width of 0.3 mm for printing microbiological disks was determined empirically to achieve the target 6 mm final disk diameter after considering hydrogel spreading upon extrusion. The filament diameter parameter in the software was kept constant at 4 mm, as the actual extruded volume was effectively controlled by the path width.

**Figure 3:**
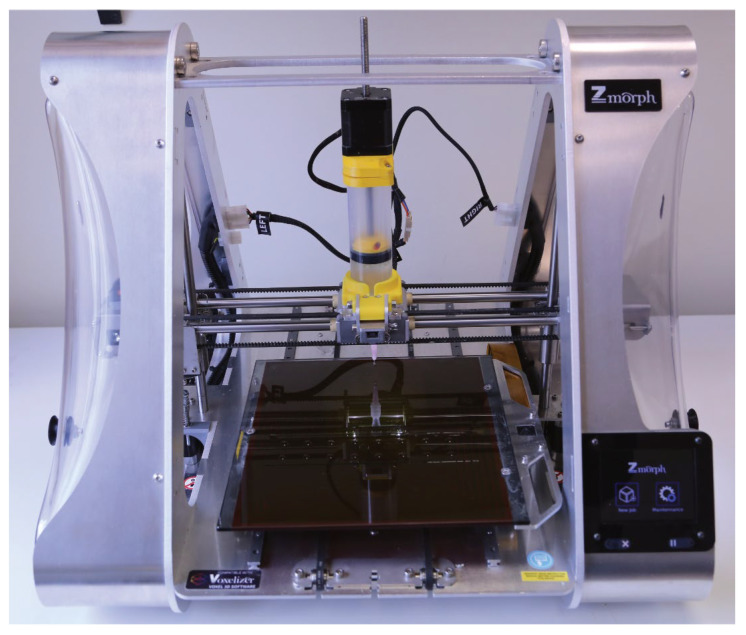
Printer configuration.

The prepared hydrogel ink was loaded into a 100 mL syringe, serving as a reservoir of printing material, and a needle was attached to the syringe outlet. The syringe was then purged of air until the needle was fully filled with the hydrogel ink and placed on the print-head carriage of the Zmorph 2.0 SX printer (Zmorph, Wrocław, Poland). Prior to printing, the printer table was leveled, and the “0” reference position on the vertical (*Z*) axis was calibrated. GCODE files were sent to the printer via Voxelizer, and printing was carried out on a clean, grease-free table (Figure 3).

**Figure 4:**
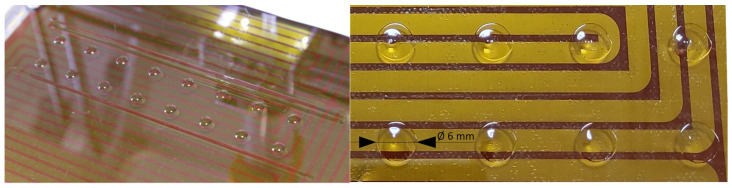
Printed microbiological disks before and after water evaporation from the hydrogel.

The printing process began and ended with the deposition of elongated hydrogel strands, which stabilized the hydrogel flow from the needle and ensured consistent printing conditions for each disk. The resulting printed microbiological disks are shown in Figure 4 (see Supplementary Materials Video S1 for the printing process).

### 2.5. Determination of Metronidazole Content in Hydrogel Inks and Disks

#### 2.5.1. Sample Preparation

To determine the MET content in printed microbiological disks and in the hydrogel inks, an appropriate sample (either a single disk or an accurately weighed amount of hydrogel ink) was placed in a volumetric flask and filled to the required volume with distilled water. The flask was then tightly sealed and placed in an ultrasonic bath at 50 °C for 30 min, allowing for complete dissolution and/or dispersion of the substance in the solution. The resulting samples were subsequently analyzed by HPLC.

#### 2.5.2. HPLC Analysis Conditions

Separation was carried out under isocratic conditions on a Synergi 4u Fusion–RP 80 Å (250 × 4.6 mm) column (Phenomenex, Torrance, CA, USA), maintained at 35 °C. The mobile phase consisted of methanol and 200 mM acetate buffer (pH 4.6) at a 15:85 (*v*/*v*) ratio, delivered at a flow rate of 1.0 mL/min. The injection volume was 40 µL, and detection was performed at a wavelength of 319 nm. The chromatographic system comprised a DGU-20A5 degasser (Shimadzu Corporation, Kyoto, Japan), two LC-20AD pumps (Shimadzu Corporation, Kyoto, Japan), an SIL-20AC autosampler (Shimadzu Corporation, Kyoto, Japan), a CTO-20AC column oven (Shimadzu Corporation, Kyoto, Japan), an SPD-M20A detector (Shimadzu Corporation, Kyoto, Japan), and a CBM-20A controller (Shimadzu Corporation, Kyoto, Japan). Data acquisition and processing were performed using LabSolutions software (version 5.101). Prior to injection, each sample was filtered through a syringe membrane filter into 1.5 mL chromatographic vials. The HPLC method for MET quantification was developed and validated in-house. Forced degradation studies confirmed the method’s specificity, showing no interference from degradation products with the metronidazole peak. The method demonstrated good linearity (R^2^ > 0.999) over the relevant analytical ranges employed in this study, and suitable limits of detection and quantification were established.

### 2.6. Microbiological Methods

#### Disk Diffusion Method

In the first stage of this study, the relationship between the MET content in the disk and the diameter of the inhibition zone in *B. fragilis* ATCC^®^ 25285™ and *F. magna* ATCC^®^ 29328™ was assessed. Metronidazole (50 µg) disks (Oxoid), normally used for antimicrobial susceptibility testing, were also tested to compare the resulting inhibition zone sizes with those produced by 3D-printed disks.

Anaerobic bacterial cultures were grown under anaerobic conditions on Brucella agar with 5% sheep blood, vitamin K, and hemin. Each plate was inoculated with either *B. fragilis* ATCC^®^ 25285™ or *F. magna* ATCC^®^ 29328™, followed by the application of 3D-printed microbiological disks. Each formulation was tested in triplicate. The plates were incubated at 37 °C using GENBag anaerobic generators (bioMérieux, Marcy-l’Étoile, France) in a Heraeus Instruments Function Line Type BB 16 CU Incubator (Heraeus, Hanau, Germany). The diameter of the inhibition zones was measured after 24 and 48 h.

In the second stage, aimed at examining the influence of hydrogel matrix components on MET activity against the selected strains, the same procedure was followed. Since *B. fragilis* ATCC^®^ 25285™ and *F. magna* ATCC^®^ 29328™ demonstrated comparable sensitivity to MET, subsequent studies were conducted exclusively with *B. fragilis* ATCC^®^ 25285™. Parallel control tests were also performed using sterile cellulose disks onto which each plasticizer (5 µL, 10 µL, 20 µL, and 50 µL) was applied, in order to evaluate the potential antimicrobial activity of the plasticizers themselves.

### 2.7. Statistical Analysis

All numerical data were expressed as mean ± standard deviation (SD). Statistical analysis was performed using TIBCO Statistica^®^ software (version 13.3, TIBCO Software Inc., Palo Alto, CA, USA). The Kruskal–Wallis test was employed to compare the diameters of bacterial growth inhibition zones for different hydrogel formulations containing MET (Stage II). A *p*-value of less than 0.05 (*p* < 0.05) was considered to indicate statistically significant differences. The sample size for the inhibition zone assays subjected to the Kruskal–Wallis test was *n* = 3 for each formulation. For MET content determination in printed disks, the number of replicates for each formulation is indicated in the respective tables.

## 3. Results

### 3.1. Determination of the Optimal Metronidazole Content in Microbiological Disks

The metronidazole content in the microbiological disks is shown in Table 1. In this experiment, the diameters of the inhibition zones noted for bacteria were measured as a function of the metronidazole (MET) concentration in the disks.

**Figure 5:**
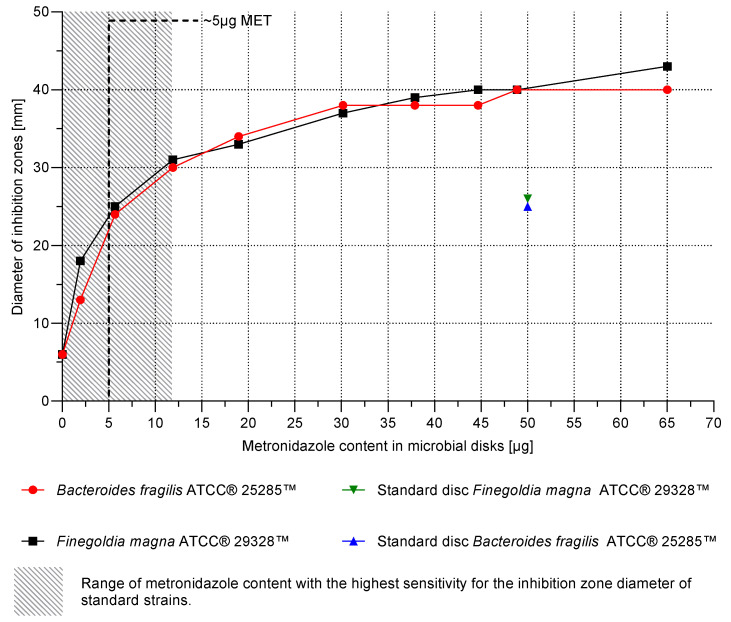
Relationship between the diameter of the inhibition zone and the metronidazole content in the standard microbiological and hydrogel disks.

A relationship between the MET content in the disks and the inhibition zone diameter was observed for both *B. fragilis* ATCC^®^ 25285™ and *F. magna* ATCC^®^ 29328™ (Figure 5). Disks containing approximately 5 µg MET were selected for subsequent analyses, as this concentration yielded the most pronounced change in inhibition zone diameter per unit of MET, thus providing the highest assay sensitivity. This MET concentration (5 µg) corresponds to the standard MET content typically used in the disk diffusion method with cellulose disks.

It was found that the 3D-printed disks containing 48.85 µg of MET exhibited an inhibition zone diameter of 40 mm, independent of the bacterial strain tested. This was larger than the zones observed for the control cellulose disks containing 50 µg of MET, which measured 25 mm for *B. fragilis* ATCC^®^ 25285™ and 26 mm for *F. magna* ATCC^®^ 29328™, as indicated by the green and blue triangles in Figure 5.

### 3.2. Determination of Metronidazole Concentration in Hydrogel Ink for Printing Microbiological Disks

**Figure 6:**
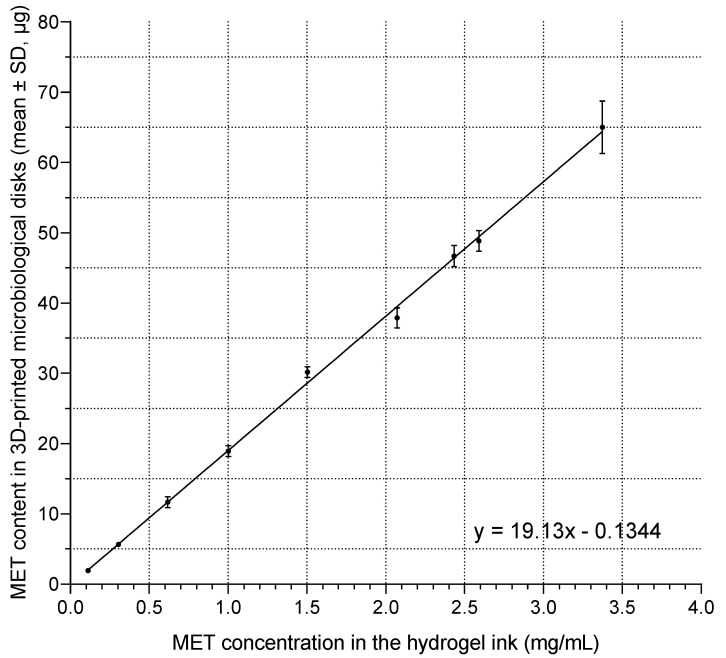
Calibration curve illustrating the correlation between MET concentration in the hydrogel ink and the resulting MET content in the 3D-printed microbiological disks. Each point represents the mean ± standard deviation (*n* = 6).

Based on the calibration curve presented in Figure 6, the concentration of MET in the hydrogel ink, necessary to obtain 3D-printed microbiological disks containing 5 µg of active substance, was determined. The optimal MET concentration in the hydrogel ink was found to be 0.27 mg/mL. The coefficient of determination (R^2^ = 0.9985) indicated a very strong linear correlation between MET concentration in the hydrogel ink and the resulting MET content in the printed disks.

### 3.3. Effect of 3D-Printed Hydrogel Matrices Composition on Antimicrobial Activity Against Bacteroides fragilis ATCC^®^ 25285™

**Figure 7:**
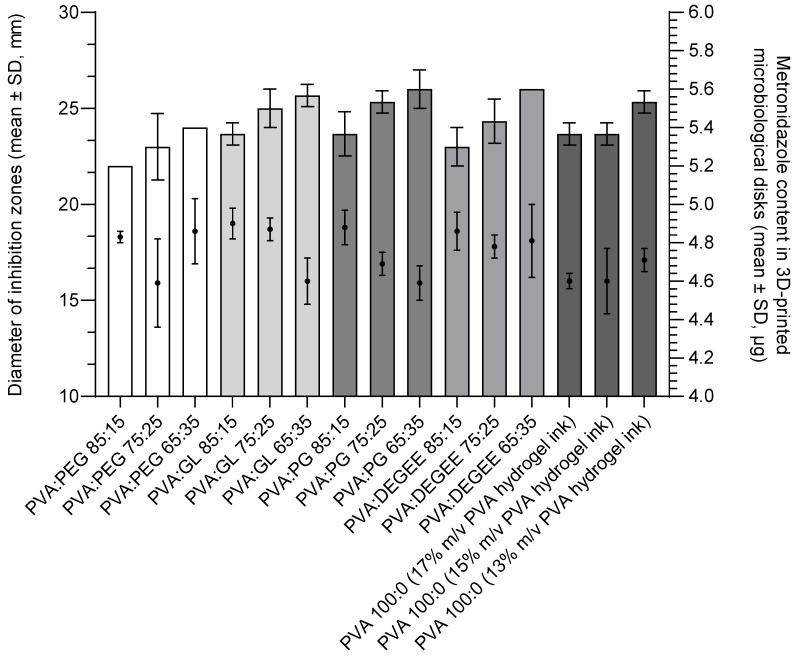
Influence of hydrogel formulation composition on metronidazole activity against *B. fragilis* ATCC^®^ 25285™ (*n* = 3); DEGEE—diethylene glycol monoethyl ether; GL—glycerol; PEG—polyethylene glycol 400; PG—propylene glycol.

In the next stage of the study, the influence of plasticizer type and content in 3D-printed microbiological disks on the antimicrobial efficacy of MET against *B. fragilis* ATCC^®^ 25285™ was evaluated. The mean MET content across all the 3D-printed microbiological disks was 4.74 µg ± 0.15 µg (detailed data on the obtained MET contents in the disks are presented in Table 4 and Figure 7).

Analysis of inhibition zone diameters indicated that the presence of plasticizers contributed positively to the antimicrobial activity of MET. In all the tested formulations, an increase in plasticizer concentration consistently resulted in larger bacterial growth inhibition zones. Additionally, similar inhibition zone diameters were observed for formulations with identical concentrations of different plasticizers in the hydrogel matrices. No reduction in MET activity was observed in any of the tested formulations.

In the plasticizer-free formulations containing 15% and 17% PVA, the average inhibition zone diameter was 23.67 ± 0.58 mm, respectively. In contrast, a larger inhibition zone (25.33 ± 0.58 mm) was observed for the formulation containing 13% PVA without any plasticizer.

Despite the observed differences in inhibition zone diameters, the Kruskal–Wallis test performed using Statistica software (version 13.3) did not show statistically significant differences (*p* > 0.05), likely due to the limited number of replicates (*n* = 3). The graphical representation of these results, illustrating the effect of hydrogel composition on inhibition zone diameter sizes, is shown in Figure 7.

### 3.4. Activity of Placebo and Individual Components of the Hydrogel Matrix

**Figure 8:**
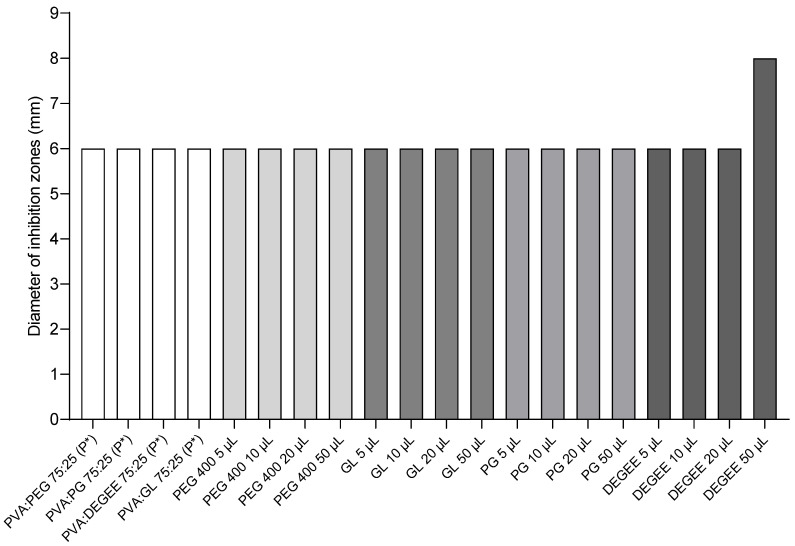
Antimicrobial activity of placebo hydrogel matrices (without API) and individual components against *B. fragilis* ATCC^®^ 25285™ (*n* = 3). The asterisk (P*) indicates a placebo formulation. An inhibition zone diameter of 6 mm corresponds to the disk diameter, indicating no antimicrobial effect on bacterial growth.

**Figure 9:**
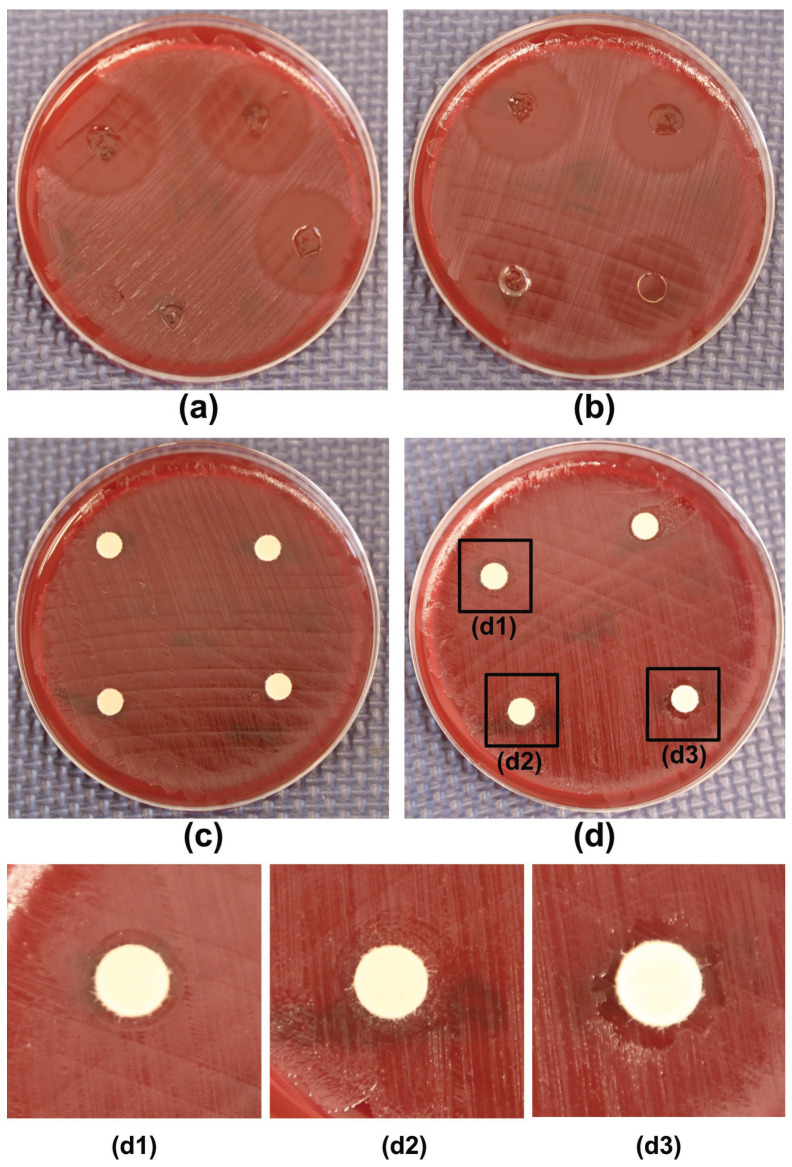
Inhibition zones of *B. fragilis* ATCC^®^ 25285™ growth for: (**a**,**b**)—seven disks containing 5 µg MET and two placebo disks; (**c**)—cellulose disks impregnated with 10 µL of plasticizers; (**d**)—cellulose disks impregnated with 50 µL of plasticizers: (**d1**)—PEG 400; (**d2**)—GC; (**d3**)—DEGEE.

In control studies, involving 3D-printed microbiological placebo disks (without MET), no inhibition zones were observed (Figure 8 and Figure 9). However, cellulose disks impregnated with plasticizers exhibited an 8 mm inhibition zone when soaked with 50 µL of DEGEE, as well as zones of partial inhibition around disks impregnated with 50 µL of PEG 400, PG, or GL (Figure 9(d1–d3)). An inhibition zone diameter of 6 mm corresponds precisely to the disk diameter, indicating no antimicrobial effect of substances released from the disk on bacterial growth.

## 4. Discussion

### 4.1. Precision and Repeatability of Metronidazole Content in 3D-Printed Microbiological Disks

One of the key advantages of 3D printing technology in pharmaceutics, including methods based on semi-solid extrusion (SSE), is the possibility of precise dosing of the active substance, which opens the way to personalization of therapy [16,17]. This technique enables controlled deposition of a specific volume of hydrogel ink containing the drug, in accordance with a digital model and precisely defined process parameters [16,17,45]. However, obtaining high dosing precision and process repeatability is conditioned by ensuring constant rheological properties of the ink (sufficient for the printing process) and optimization of the printing parameters themselves [16,45]. Ink inhomogeneity, changes in viscosity, or the presence of air bubbles can lead to irregular flow of the material through the nozzle, which results in variability in the mass of the extruded material and thus the drug dose in the final printout [16,17,45]. Nevertheless, as other studies show, with appropriate control of the formulation and printing process, it is possible to produce drug forms with very good uniformity of the active substance content [17,40].

In the context of this work, the possibility of precise MET dosing using SSE 3D printing was confirmed in the adopted two-stage research approach.

The first stage of the research was conducted to determine the optimal MET content in the printed disks. The aim was not only to obtain control over the amount of the drug but, above all, to identify the MET content that would provide the highest sensitivity of the disk diffusion method in the subsequent assessment of the effect of plasticizers on drug activity (Stage II). For this purpose, a series of hydrogel inks with different, strictly controlled MET concentrations were prepared, from which microbiological disks were then printed. These disks were subjected to MET content analysis and evaluation in the disk diffusion test against *B. fragilis* to determine at what content of the active substance the greatest changes in the diameter of the zones of inhibition of bacterial growth are obtained.

At the same time, the analysis of MET content in the disks confirmed the possibility of precise control of the drug dose. A strong, linear relationship (R^2^ = 0.9985) was demonstrated between the drug concentration in the hydrogel ink and the final MET content in a single printed disk (Figure 6). This high correlation proved the predictability of the printing process and allowed for the precise determination of the MET concentration in the ink (0.27 mg/mL), which allowed for obtaining a target dose of 5 µg in the disk. This dose was selected for the second stage of the study, because, as it resulted from the microbiological analysis (Figure 5), it provided the highest sensitivity of the method (the largest changes in the zone diameter for small changes in MET content).

Already at this stage, the analysis of MET content in printed disks from individual formulations (Table 1) showed good within-batch precision, characterized by low relative standard deviation (RSD) values, mostly below 6%, which confirmed the repeatability of prints achieved thanks to controlled printing conditions [40].

The second stage of this study allowed for the assessment of the repeatability and precision of MET content for formulations differing in composition. Using the hydrogel ink with the MET concentration optimized in Stage I, a series of microbiological disks were produced, differing in the type and concentration of the added plasticizer (PEG 400, GL, PG, DEGEE). The analysis of MET content in all disks obtained in this stage (Table 4) showed that the average content of the active substance was 4.74 µg ± 0.15 µg and was close to the declared dose of 5 µg (94.8 ± 3% DV of declared value), which indicates the high accuracy of the printing process. In addition, the low standard deviation and low relative standard deviation (RSD below 3.2%), calculated for all Stage II disks, regardless of the type and amount of plasticizer, clearly confirm the high precision and repeatability of the dosing process, achievable thanks to the applied 3D printing methodology. The obtained level of precision indicates very good process control, comparable or exceeding the levels of mass or content uniformity achieved in other studies on SSE printing, where problems related to the lack of repeatability of prints often occur [17,45]. The low variability of MET content between different formulation series obtained in this study proves that the developed method is resistant to small changes in the ink composition (in the range of tested plasticizers and polymers), which is important from the point of view of potential standardization of this method. Furthermore, the precision within the series for individual formulations in Stage II was also very high, with RSD values often below 2% (Table 4). This is particularly noteworthy in the context of the study by Díaz-Torres et al., which presented significant variability and difficulties in obtaining precise doses in the range of 2–6 mg of hydrochlorothiazide. They attributed this to the small volume of extruded material and the greater influence of process errors at such small quantities [17]. In contrast, the present study showed that high reproducibility could be achieved for a content of 5 µg MET.

### 4.2. Manufacturing of Microbiological Disks Using 3D Printing: A Standardized Platform

The disk diffusion method is the standard for assessing microbial susceptibility and uses cellulose disks impregnated with an antimicrobial solution. Although this method is widely accepted, the literature indicates its limitations when evaluating more complex drug delivery systems such as hydrogel dressings. Non-uniformity of impregnation, differences in drug adsorption in the hydrogel to cellulose, and different release kinetics from the cellulose disk compared to the target polymer matrix can lead to variability in results and difficulties in their interpretation. In response to these challenges, and to establish a more relevant and reproducible screening tool, this work developed and applied a method for producing standardized microbiological disks based on PVA hydrogel using SSE 3D printing.

The advantage of using 3D printing in this context is the precise control over the amount of active substance (MET) and the composition of the matrix material contained in each printed disk. As demonstrated in Stage II of this study, the developed method is characterized by very high repeatability of MET content. In contrast to the potential variability associated with manual impregnation of cellulose disks, 3D printing allows for automated production of uniform test carriers. Moreover, the use of a PVA hydrogel matrix as the disk material allows for better imitation of the target drug delivery system (e.g., a hydrogel dressing) at the pre-formulation or formulation screening stage. MET diffusion from the PVA matrix to the agar substrate during a microbiological test may more realistically reflect the drug release process from the dressing than diffusion from an impregnated cellulose disk. This allows for the assessment of not only the activity of the active substance itself, but also the effect of the matrix formulation (e.g., the presence of plasticizers) on its availability and diffusion under test conditions, which is a key aspect of the developed platform.

In the literature on 3D-printed antimicrobial drug delivery systems, the assessment of microbiological activity is often performed by directly placing the printed material on an inoculated agar substrate [46,47,48,49,50,51,52]. These methods are suitable for evaluation of the final therapeutic product but may be less useful for standardized comparison of the effect of subtle formulation changes on drug activity, due to variability in scaffold geometry and surface area. Our method, using precisely defined, printed disks as a test tool, offers an alternative focused on reproducibility and standardization for the microbiological evaluation of different drug-eluting matrix material formulations, serving as an early-stage screening platform.

### 4.3. Effect of Plasticizers on Metronidazole Activity and Potential Underlying Mechanisms

A central aim of utilizing the developed 3D printing platform was to investigate the influence of different plasticizers on the antimicrobial activity of MET. The obtained results indicate that the tested PVA-based hydrogel formulations, including those containing plasticizers at the used concentrations, did not show a reduction in the antibacterial efficacy of MET against *B. fragilis*. The growth inhibition zones around the disks obtained using pure PVA are of comparable size to those observed for disks containing the selected plasticizers. This suggests that the interactions in these systems, both between PVA and MET and between plasticizers and MET, are mainly physical in nature and do not lead to unfavorable chemical interactions, degradation, or inactivation of the drug. Studies confirming the compatibility of MET with PVA-based systems were conducted by various authors using thermal and spectroscopic methods. Thermal analysis by differential scanning calorimetry (DSC), both in hydrogels containing PVA and gelatin [53] and in semi-crystalline PVA matrices [54], showed no significant chemical or thermal interactions between MET and the polymer matrix. Moreover, Fourier-transform infrared spectroscopy (FTIR) analysis confirmed the presence of characteristic drug peaks in the final cross-linked polymer, which further indicates good compatibility of both components [53].

DEGEE is widely used in dermatological preparations as a solvent and permeation promoter, known for its good safety and tolerability profile; the literature does not indicate its significant chemical reactivity with typical drug substances [44]. Similarly, glycerol, which is a commonly used humectant and plasticizer, is considered chemically inert in most formulations [55,56]. Many studies use PG, PEG 400, or GL as components of MET-containing formulations, focusing on their effect on solubility, drug release rate, or its penetration through biological barriers, without reporting problems related to chemical incompatibility [39,57,58,59,60]. These observations, combined with our results indicating the preservation of full antimicrobial activity of MET in the presence of all tested plasticizers, support the thesis of good chemical compatibility of MET with PG, GL, PEG 400, and DEGEE within the developed PVA-based hydrogel system.

Of note, a small, although statistically insignificant (probably due to the limited number of replicates, n = 3, for microbiological tests), trend towards an increase in the diameter of the growth inhibition zones with increasing plasticizer concentration in the formulations was observed (Figure 7). The observed antimicrobial activity in a disk diffusion assay is a function of both the intrinsic potency of the drug and its ability to diffuse from the delivery system into the agar medium. Therefore, any influence of plasticizers on MET’s apparent activity in this system is likely mediated through their impact on the hydrogel matrix properties, which in turn govern the drug’s release and subsequent diffusion. This trend is consistent with reports in the literature indicating that plasticizers can facilitate or accelerate the release of drugs from polymer matrices. Several mechanisms could contribute to this:

- Modification of matrix structure and crystallinity: Plasticizers, such as those used in this study, are known to modify the structure of PVA-based hydrogels. They can reduce the degree of crystallinity of PVA matrices and alter thermal properties like the glass transition temperature (T_g_) and melting temperature of crystallites, often by increasing polymer chain mobility, disrupting polymer chain interactions, or destroying hydrogen bonds between macromolecules [33,61]. For instance, Mohammed and El-Sayed demonstrated that the addition of PEG (Mw 4000) significantly decreased the crystallinity (X_c_) from 31.24% to 25.45% and lowered the T_g_ of PVA films from 89.2 to 60.6 °C [62]. Similarly, Panova et al. reported that the addition of glycerol to PVA films led to a significant decrease in T_g_ (from 94 °C for pure PVA to 42 °C for PVA with 20 wt% glycerol) and a reduction in the degree of crystallinity (from 49% for pure PVA to 42% for PVA with 20 wt% glycerol) [61]. A less ordered, more amorphous polymer structure can facilitate drug diffusion through the matrix and counteract the retarding effect of high crystallinity on drug release, as observed for MET by Mallapragada et al. [54].
- Enhancement of diffusion channels and swelling: Hydrophilic plasticizers can enhance the hydration of the hydrogel matrix, potentially leading to the enlargement of diffusion channels within the polymer network when in contact with the aqueous environment of the agar [56]. This increased free volume and matrix porosity can accelerate the drug release rate. For example, PEG has been used as a porogen to improve the permeability and mass transfer capability of PVA hydrogels; its addition led to the formation of pores within the gel, providing channels for diffusion, with larger pores forming at higher PEG molecular weights or concentrations [63]. Studies by Abdel-Mottaleb et al. on PVA hydrogels also showed that the addition of PEG (including PEG 400) significantly increased the release rate of fluconazole [64].
- Co-solvency or improved drug dispersion: Plasticizers can act as co-solvents or improve the dispersion of the API within the hydrogel matrix [41,58]. The plasticizers used in this study (DEGEE, glycerol, PEG 400, and propylene glycol) are known for their solvent properties. DEGEE is recognized as a powerful solubilizing agent and penetration enhancer [44,59], and PG has been shown to increase the solubility of metronidazole in topical formulations [58,59]. For example, Lee et al. demonstrated that PEG 400 was an excellent solvent for lifitegrast, significantly increasing its solubility compared to water, and these solvents (DEGEE, GL, PEG 400, PG) are generally recognized as “green solvents” [65]. Enhanced solubilization or finer dispersion of MET within the PVA matrix due to the presence of these plasticizers could lead to a higher effective concentration gradient at the disk–agar interface, thereby promoting more efficient drug transport into the agar, in accordance with the observations of Orienti et al. regarding the effect of solubilization on drug release [66]. Cai et al. also observed that glycerol accelerated insulin release, especially in the initial phase from PVA hydrogels [56]. Studies on the release of ciprofloxacin from PVA matrices also showed that the addition of PEG 6000 significantly increased the release efficiency, with proposed mechanisms including an increase in microchannel size and number and a weakening of PVA–drug interactions [67].

While detailed drug release studies from these specific printed disks were beyond the scope of this initial platform validation, the observed trends in inhibition zones with varying plasticizer concentrations are consistent with such modulation of drug availability. This suggests that the developed 3D printing platform is sensitive enough to detect subtle formulation-dependent variations, warranting further investigation into precise release kinetics in future studies focused on optimizing final dosage forms.

It is also worth noting the results of the control studies. Three-dimensionally printed placebo disks (without MET) did not show growth inhibition zones (Figure 8 and Figure 9), suggesting a lack of intrinsic antimicrobial activity of the hydrogel matrix itself under the conditions used. However, cellulose disks impregnated with pure plasticizers (50 µL) showed some activity: an inhibition zone of 8 mm was observed for DEGEE (Figure 9), as well as zones of partial inhibition for PEG 400, PG, and GL (Figure 9(d1–d3)). The observed activity of pure DEGEE is interesting, as the literature indicates that DEGEE generally does not show strong antimicrobial properties against tested standard strains, although mild inhibition of anaerobic *Propionibacterium acnes* has been previously reported [44]. It is possible that the observed growth inhibition zones are due to the high concentration of plasticizers on the cellulose disk (e.g., effect of high osmotic pressure) and, additionally, in the case of DEGEE, the specific sensitivity of the *B. fragilis* strain to this substance. However, the key fact is that this activity did not occur in the case of placebo hydrogel disks (containing the same plasticizers in the PVA matrix). This suggests that the concentrations of plasticizers released from the hydrogel matrix are too low to cause an inhibitory effect on their own, or their availability is strongly modulated by the PVA matrix. This confirms that the observed antimicrobial activity of the drug disks is primarily due to the action of released MET.

An important and somewhat unexpected result of the study was the observation that 3D-printed hydrogel disks containing approximately 50 µg of MET showed significantly larger growth inhibition zones for *B. fragilis* and *F. magna* than standard commercial cellulose disks with the same nominal drug content (Figure 5). Explaining this phenomenon requires considering several potential factors. First, the release kinetics of MET from the hydrogel matrix may differ from the release kinetics from the cellulose disk, potentially being more favorable from the hydrogel. Second, the hydrated hydrogel matrix may provide better contact between the disk and the agar matrix, facilitating MET diffusion into the substrate. Third, the physical state and degree of dispersion of MET in the PVA matrix with plasticizers may be important. As discussed earlier, plasticizers can act as cosolvents [41,58,65,66], potentially leading to better dispersion or even partial solubilization of MET in the hydrogel matrix. In contrast to the standard cellulose disk, in which the drug is in a crystalline form (requiring dissolution before diffusion), better dispersion in the hydrogel may result in a higher effective concentration gradient at the disk–agar interface. Such an increased gradient would enhance diffusion, leading to more efficient drug transport into the agar matrix and, as a result, larger zones of bacterial growth inhibition. However, further dedicated studies, including a detailed comparative analysis of MET release profiles from both types of carriers, would be needed to confirm the dominant mechanism.

## 5. Study Limitations and Challenges

While this study successfully demonstrates the development and utility of a 3D printing platform for producing standardized MET-loaded hydrogel disks and evaluating the impact of plasticizers on MET content uniformity and in vitro antimicrobial activity, certain limitations should be acknowledged. It is important to note that this study aimed to validate a research platform, and therefore, certain analyses typical for late-stage product development were not within its immediate scope.

It should be emphasized that the presented investigation was conducted in vitro, and its primary focus was on establishing the 3D printing methodology as a platform for assessing fundamental formulation attributes—specifically API content and antimicrobial activity. Consequently, aspects crucial for the development of a final therapeutic dressing, such as a detailed analysis of MET release kinetics from the hydrogel matrices, their long-term stability, comprehensive characterization of mechanical and rheological properties, bioadhesion, or in vivo efficacy and biocompatibility, were beyond the defined objectives of this particular study. These crucial analyses are envisioned as subsequent steps in the broader project of developing a clinically viable wound dressing, for which the current platform provides a foundational screening tool. Similarly, the assessment of antimicrobial activity, while demonstrating the platform’s utility, was ultimately focused on a single strain of *B. fragilis* for the comparative evaluation of plasticizer effects and did not extend to a wider range of wound pathogens.

Moreover, in some comparative microbiological assessments (e.g., differences in inhibition zone sizes between formulations with different plasticizers), the number of replicates (n = 3) was limited, which may have influenced the statistical power to detect subtle differences. The conclusions regarding plasticizer compatibility are specific to the PVA-MET system we investigated and may not be directly transferable to other polymer–drug combinations without further validation using the platform. The potential influence of sterilization methods on the hydrogel properties and drug activity, a critical consideration for wound care products, was also not addressed within the scope of this platform-focused study and remains an important area for future investigation in the context of product development.

The results and limitations of this work indicate several promising directions for further research. Building upon the validated platform, future studies should include in vivo assessments on infected animal wound models to evaluate the therapeutic efficacy of hydrogels developed using insights from this platform. The extension of microbiological studies to include other clinically relevant pathogens (aerobic, anaerobic) and to assess the activity against bacterial biofilms is also necessary. Detailed investigations into MET release kinetics, long-term stability (of both MET in the matrix and the hydrogels themselves under various conditions, including the influence of plasticizers and potential sterilization), full mechanical and rheological characterization, and comprehensive biocompatibility testing (in vitro and in vivo) are crucial for translating these findings into a practical therapeutic application. Further optimization of the formulation (e.g., PVA concentration, cross-linking, alternative excipients) and the printing process, as well as the design of complex, personalized dressing geometries, represent other important avenues for future development.

## 6. Conclusions

This study successfully developed and validated an SSE 3D printing technique as a robust and reproducible platform for manufacturing standardized PVA-based hydrogel disks loaded with MET. The primary achievement is the establishment of these 3D-printed disks as a reliable tool for the systematic and standardized evaluation of excipient impact on API characteristics, particularly content uniformity and antimicrobial performance, which was the central hypothesis of this work.

Key findings demonstrated that the 3D printing platform enables the production of hydrogel disks with high precision and repeatability of MET content, a critical factor for reliable comparative studies. Utilizing this platform, it was shown that the incorporation of selected plasticizers (polyethylene glycol 400, glycerol, propylene glycol, and diethylene glycol monoethyl ether) into the PVA-MET hydrogel formulation did not adversely affect the antimicrobial efficacy of MET against *Bacteroides fragilis*. This addresses the study’s objective concerning the influence of these excipients. Furthermore, the 3D-printed hydrogel disks exhibited antimicrobial activity comparable to, or in some instances greater than, standard cellulose disks with similar MET loading, highlighting the potential of the platform for generating relevant comparative data for formulation screening.

In conclusion, the developed SSE 3D printing platform offers a valuable and optimized methodology for preformulation studies and excipient screening within a Quality by Design framework. It facilitates a more rational approach to designing and developing hydrogel-based drug delivery systems by allowing for controlled investigation of how formulation variables influence critical quality attributes of the API. This work, using MET as a model drug and plasticizers as model excipients, underscores the platform’s potential utility in the broader context of pharmaceutical development, including the future design of personalized topical medications and as a tool to better understand drug–excipient interactions in 3D-printed dosage forms.

## Figures and Tables

**Table 1:** Amounts of metronidazole (MET) used in Stage I and final MET content in the 3D-printed microbiological disks (*n* = 6), intended to determine the optimal antimicrobial load. All hydrogel ink formulations contained 7.5 g PVA, 2.5 g PEG 400, and water up to 50 mL; RSD—relative standard deviation; SD—standard deviation.

Formulation	MET Content in Hydrogel (mg)	MET Content in Disk (mean ± SD, µg)	RSD of MET Content in Disk (%)
1	0	0.00	0
2	5.62	1.93 ± 0.08	4.1
3	15.22	5.66 ± 0.30	5.4
4	30.85	11.68 ± 0.77	6.6
5	50.07	18.94 ± 0.78	4.1
6	75.18	30.17 ± 0.78	2.6
7	103.6	37.89 ± 1.42	3.8
8	121.65	46.67 ± 1.51	3.2
9	129.54	48.85 ± 1.48	3.0
10	168.68	65.02 ± 3.71	5.7

**Table 2:** Formulations of hydrogel inks used in Stage II.

Formulation	Concentration of Substances in the Hydrogel Inks for 3D Printing						
	PVA–Plasticizer Ratio	PVA (% *m*/*v*)	PEG 400 (% *m*/*v*)	PG (% *m*/*v*)	DEGEE (% *m*/*v*)	GL (% *m*/*v*)	MET (% *m*/*v*)
PVA–PEG 85:15	85:15	17.0	3.0	–	–	–	0.027
PVA–PEG 75:25	75:25	15.0	5.0	–	–	–	0.027
PVA–PEG 65:35	65:35	13.0	7.0	–	–	–	0.027
PVA–GL 85:15	85:15	17.0	–	–	–	3.0	0.027
PVA–GL 75:25	75:25	15.0	–	–	–	5.0	0.027
PVA–GL 65:35	65:35	13.0	–	–	–	7.0	0.027
PVA–PG 85:15	85:15	17.0	–	3.0	–	–	0.027
PVA–PG 75:25	75:25	15.0	–	5.0	–	–	0.027
PVA–PG 65:35	65:35	13.0	–	7.0	–	–	0.027
PVA–DEGEE 85:15	85:15	17.0	–	–	3.0	–	0.027
PVA–DEGEE 75:25	75:25	15.0	–	–	5.0	–	0.027
PVA–DEGEE 65:35	65:35	13.0	–	–	7.0	–	0.027
PVA 100:0 (17% *m*/*v* PVA hydrogel ink)	100:0	17.0	–	–	–	–	0.027
PVA 100:0 (15% *m*/*v* PVA hydrogel ink)	100:0	15.0	–	–	–	–	0.027
PVA 100:0 (13% *m*/*v* PVA hydrogel ink)	100:0	13.0	–	–	–	–	0.027
PVA–PEG 75:25 (P *)	75:25	15.0	5.0	–	–	–	–
PVA–PG 75:25 (P *)	75:25	15.0	–	5.0	–	–	–
PVA–DEGEE 75:25 (P *)	75:25	15.0	–	–	5.0	–	–
PVA–GL 75:25 (P *)	75:25	15.0	–	–	–	5.0	–

* Placebo hydrogel.

**Table 3:** Printing parameters for microbiological disks (Voxelizer settings).

Parameter	Value
Filament diameter	4 mm
Layer count	1
Layer height	0.1 mm
Path width	0.3 mm
Travel speed	120 mm/s
Print speed	10 mm/s
Extrusion order	Nearest (proximity)
Retraction	Enabled
Retraction height	2 mm
Retraction amount	0 mm
Retraction speed (*Z* axis)	50 mm/s
Retraction speed (extrusion)	50 mm/s
Retraction minimum distance	10 mm
Extra length on restart	0 mm
Needle size	Diameter: 1.2 mm, length: 5 mm, flat-cut
Build platform temperature	Room temperature
Drying time and conditions	24 h, room temperature
Airflow	Disabled

**Table 4:** Measured metronidazole (MET) content (*n* = 3 per formulation) and precision in 3D-printed microbiological disks with different plasticizers (Stage II); % DV—percentage of declared value; RSD—relative standard deviation; SD—standard deviation.

Formulation	MET Content (mean ± SD, µg)	RSD (%)	% DV (mean ± SD, %)
PVA–PEG 85:15	4.83 ± 0.03	0.62	96.6 ± 0.6
PVA–PEG 75:25	4.59 ± 0.23	5.01	91.8 ± 4.6
PVA–PEG 65:35	4.86 ± 0.17	3.50	97.2 ± 3.4
PVA–GL 85:15	4.90 ± 0.08	1.63	98.0 ± 1.6
PVA–GL 75:25	4.87 ± 0.06	1.23	97.4 ± 1.2
PVA–GL 65:35	4.60 ± 0.12	2.61	92.0 ± 2.4
PVA–PG 85:15	4.88 ± 0.09	1.84	97.6 ± 1.8
PVA–PG 75:25	4.69 ± 0.06	1.28	93.8 ± 1.2
PVA–PG 65:35	4.59 ± 0.09	1.96	91.8 ± 1.8
PVA–DEGEE 85:15	4.86 ± 0.10	2.06	97.2 ± 2.0
PVA–DEGEE 75:25	4.78 ± 0.06	1.26	95.6 ± 1.2
PVA–DEGEE 65:35	4.81 ± 0.19	3.95	96.2 ± 3.8
PVA 100:0 (17% *m*/*v* PVA hydrogel ink)	4.60 ± 0.04	0.87	92.0 ± 0.8
PVA 100:0 (15% *m*/*v* PVA hydrogel ink)	4.60 ± 0.17	3.70	92.0 ± 3.4
PVA 100:0 (13% *m*/*v* PVA hydrogel ink)	4.71 ± 0.06	1.27	94.2 ± 1.2

## Data Availability

The original contributions presented in this study are included in the article/Supplementary Material. Further inquiries can be directed to the corresponding author.

## Supplementary Materials

The following supporting information can be downloaded at: https://www.mdpi.com/article/10.3390/pharmaceutics17060749/s1, Video S1: 3D Printing of hydrogel disks.

## Abbreviations

The following abbreviations are used in this manuscript:

API	active pharmaceutical ingredient
ATCC	American Type Culture Collection
DEGEE	diethylene glycol monoethyl ether (Transcutol^®^ P)
DSC	differential scanning calorimetry
DV	declared value
FTIR	Fourier-transform infrared spectroscopy
GL	Anhydrous glycerol
HFE	hydrogel-forming extrusion
HPLC	high-performance liquid chromatography
MET	metronidazole
PEG 400	polyethylene glycol 400
PG	propylene glycol
Ph. Eur.	European Pharmacopoeia
PVA	polyvinyl alcohol 31,000–50,000, 98–99% hydrolyzed
R^2^	coefficient of determination
RSD	relative standard deviation
SD	standard deviation
SSE	semi-solid extrusion
